# The Effectiveness of Transcranial Magnetic Stimulation (TMS) Paradigms as Treatment Options for Recovery of Language Deficits in Chronic Poststroke Aphasia

**DOI:** 10.1155/2022/7274115

**Published:** 2022-01-11

**Authors:** Anastasios M. Georgiou, Maria Kambanaros

**Affiliations:** ^1^The Brain and Neurorehabilitation Lab, Department of Rehabilitation Sciences, Cyprus University of Technology, Cyprus; ^2^Allied Health and Human Performance, University of South Australia, Adelaide, Australia

## Abstract

**Background:**

In an effort to boost aphasia recovery, modern rehabilitation, in addition to speech and language therapy (SALT), is increasingly incorporating noninvasive methods of brain stimulation. The present study is aimed at investigating the effectiveness of two paradigms of neuronavigated repetitive transcranial magnetic stimulation (rTMS): (i) 1 Hz rTMS and (ii) continuous theta burst stimulation (cTBS) each as a standalone treatment for chronic aphasia poststroke.

**Methods:**

A single subject experimental design (SSED) trial was carried out in which six people with aphasia (PWA) were recruited, following a single left hemispheric stroke more than six months prior to the study. Three individuals were treated with 1 Hz rTMS, and the remaining three were treated with cTBS. In all cases, TMS was applied over the right pars triangularis (pTr). Language assessment, with standardized and functional measures, and cognitive evaluations were carried out at four time points: twice prior to treatment (baseline), one day immediately posttreatment, and at follow-up two months after treatment was terminated. Quality of life (QoL) was also assessed at baseline and two months posttreatment. In addition, one of the participants with severe global aphasia was followed up again one and two years posttherapy.

**Results:**

For all participants, both rTMS paradigms (1 Hz rTMS and cTBS) generated trends towards improvement in several language skills (i.e., verbal receptive language, expressive language, and naming and reading) one day after treatment and/or two months after therapy. Rated QoL remained stable in three individuals, but for the other three, the communication scores of the QoL were reduced, while two of them also showed a decline in the psychological scores. The participant that was treated with cTBS and followed for up to two years showed that the significant improvement she had initially exhibited in comprehension and reading skills two months after TMS (1^st^ follow-up) was sustained for at least up to two years.

**Conclusion:**

From the current findings, it is suggested that inhibitory TMS over the right pTr has the potential to drive neuroplastic changes as a standalone treatment that facilitates language recovery in poststroke aphasia.

## 1. Introduction

To boost poststroke aphasia rehabilitation further, several noninvasive brain stimulation (NIBS) techniques have been applied to poststroke aphasia individuals over the past 20 years with promising results. Two of the most common methods that are being investigated are transcranial magnetic stimulation (TMS) and transcranial direct current stimulation (tDCS). The rationale behind their application is that both methods modulate neuronal plasticity and, in this way, facilitate language recovery.

Transcranial magnetic stimulation has shown exploratory potential to induce language recovery in aphasia poststroke [1]. Before 2014, only a few rTMS studies on poststroke aphasia recruited sufficiently large numbers of participants [2]. The majority of those studies explored the effects of low-frequency (LF) TMS over the contralesional inferior frontal gyrus (IFG) followed by speech and language therapy (SALT) in a clinically heterogeneous group of people with aphasia (PWA) at the postacute phase of recovery [3–6] with mixed results; hence, no conclusions could be drawn regarding the efficacy of LF rTMS over the contralesional IFG on recovery of poststroke aphasia [2]. After 2014, additional research with larger numbers of PWA has offered further insight on the possible effectiveness of rTMS on aphasia recovery in subacute aphasia [7, 8] and in the chronic stage [9–11]. The potential positive outcomes of rTMS on aphasia recovery poststroke have been further investigated by trials applying short rTMS burst protocols (e.g., theta burst stimulation (TBS)) with promising results [12–16]. For a review of TBS, see Huang and Rothwell [17] and Huang et al. [18]. Collective findings from LF rTMS in poststroke aphasia suggest that LF rTMS over the right IFG has the potential to reorganize the language networks and drive language improvement in people with poststroke aphasia. Nevertheless, with regard to high-frequency (HF) TMS, according to a recent review [2], no recommendations can be made for its use in poststroke aphasia rehabilitation.

Research on TMS aphasia rehabilitation is ongoing and promising but remains inconclusive for several reasons. For example, there are many inconsistencies between studies in several domains such as the following: (i) number of participants, (ii) paradigms employed (inhibitory vs. excitatory rTMS and inhibitory together with excitatory rTMS), (iii) anatomical sites of stimulation, (iv) methods of localization of stimulation sites (e.g., 10-20 international system vs. frameless stereotactic neuronavigation systems), (v) type and intensity of SALT, and (vi) the use of reliable outcome measures. With regard to SALT that is used as adjuvant to TMS, there are several studies that highlight major inconsistencies in SALT types and intensities. Examples of relevant randomized controlled trials include a study [7] in which a 45-minute SALT program was applied according to best-practice guidelines [19], a trial [20] in which a 30-minute SALT program focusing on language comprehension and expression was followed, a study [21] that used a 30-minute SALT regimen focusing on naming, another study [8] that applied a 45-minute SALT program aimed at reactivation of word retrieval, another trial [11] that used a 60-minute SALT program twice a week emphasising verbal expressive skills, five trials that followed a 45-minute SALT program focused on patient-specific language problems [3–5, 22, 23], and a study [6] that applied a 45-minute program focusing on expression and comprehension of spoken language. The wide variability in the reported studies and the absence of standardization of the SALT programs question their efficacy by not allowing the disentanglement of the beneficial effects of TMS from those of SALT. Therefore, the extent of the improvement on language abilities attributed to TMS cannot be evaluated.

The present study is aimed at measuring the effectiveness of rTMS as a standalone treatment for chronic stroke-induced aphasia. The objectives of the study were as follows:

- To explore whether continuous 1 Hz rTMS and cTBS (independent variables, IV) could modify performance on language tests (dependent variables, DV) one day (short-term) and/or two months (long-term) posttreatment when administered for 10 consecutive days over the right pars triangularis (pTr) of individuals with chronic aphasia
- To explore whether the above protocols (i.e., cTBS and 1 Hz rTMS) could bring about similar changes in language performance in the cohort of PWA under investigation

## 2. Materials and Methods

### 2.1. Bioethics Approval

Ethical approval for this study was obtained from the Cyprus National Bioethics Committee (CNBC) (EEBK/E*Π*/2017/37).

### 2.2. Participants

**Figure 1:**
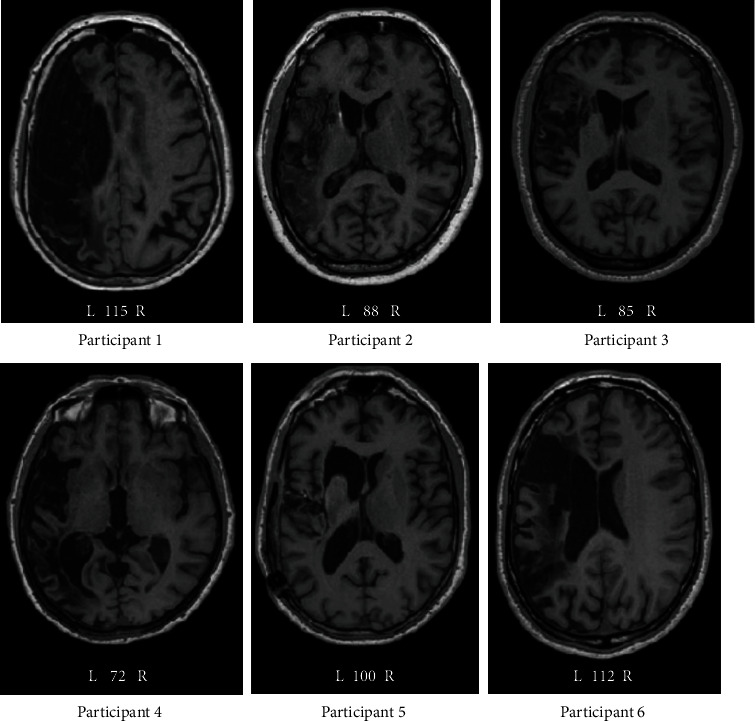
Brain MRI scans (axial plane) of the participants. Key: L: left hemisphere; R: right hemisphere; numbers indicate serial axial slice images.

A single-subject experimental design (SSED) trial was undertaken at the University Rehabilitation Clinic of the Department of Rehabilitation Sciences at the Cyprus University of Technology (CUT). Adults who had suffered a single left hemisphere stroke at least six months prior to participating in the study were actively sought for recruitment. The recruitment phase was open for 15 months. The inclusion criteria were as follows: (1) aged between 18 and 75 years of age, (2) native speakers of (Cypriot) Greek, (3) right-handed, (4) a diagnosis of a first ever left-sided middle cerebral artery (MCA) stroke verified by magnetic resonance imaging (MRI) or computerized tomography (CT), (5) chronic aphasia stage (>6 months poststroke), (6) no history of dementia or other neurological illnesses, and (7) no current participation in any type of language rehabilitation. Exclusion criteria included the following: (1) Greek not the mother tongue; (2) left-handedness; (3) prior stroke(s); (4) MRI and TMS exclusion criteria; (5) severe dysarthria affecting intelligibility; (6) any other neurological condition affecting the sensorimotor system (e.g., brain tumour); (7) medication that alerts brain excitability to avoid pharmacological influences on TMS, as there is evidence that the extent and direction of NIBS-induced plasticity can be significantly modulated by many neuropharmacological agents [24]; (8) cognitive disorders known before the stroke; and (9) involvement in behavioral language rehabilitation. Overall, 20 people were recruited but only eight actively took part and completed all phases of the study. Two participants were recruited to the pilot study (see [25]) and the remaining six to the main study (see Table 1 for demographics and clinical characteristics of the six participants and Figure 1 for brain MRIs). The remaining seven individuals from the initial cohort did not participate due to caregivers' reluctance/refusal because of time commitment to the study, and three PWA withdrew from the study during the TMS treatment stage while two more withdrew because of claustrophobia and subsequent failure to undergo an MRI scan.

### 2.3. Study Eligibility Measures

To determine eligibility for the study the following measures were carried out: (1) a detailed case history on demographics and health status, (2) a screening checklist for TMS eligibility, (3) the Hemispatial Neglect Test [26], and (4) the Handedness Inventory (Edinburgh Handedness Inventory—Short Form [27]) to determine handedness.

### 2.4. Cognitive-Linguistic Measures Performed at Baseline, Posttreatment, and Follow-Up

#### 2.4.1. The Greek Boston Diagnostic Aphasia Examination-Shortened Version (BDAE-SF)

The Greek BDAE-SF [28] was used for language examination (i.e., oral and written language comprehension, expressive language, reading, and writing).

#### 2.4.2. The Peabody Picture Vocabulary Test–Revised (PPVT-R)

The short form (32 stimuli) of the Greek PPVT-R [29] was used to measure single word receptive vocabulary. The full and short versions of the PPVT-R are equivalent and constitute reliable and valid assessment tools of vocabulary for Greek students and immigrants who speak Greek [29].

#### 2.4.3. The Greek Object and Action Test (GOAT)

The Greek Object and Action Test (GOAT) is used to assess naming of nouns and verbs for assessment and/or research purposes in Greek speakers. It contains 84 coloured photographs measuring 42 actions and 42 objects. The test in total (production and comprehension subtests) takes under an hour to administer. The GOAT is reported in published studies investigating verb-noun grammatical dissociations across language-impaired populations [30]. For the purposes of this study, 19 informative verbs were used that distinguish language impaired from nonimpaired groups. This informative version was produced based on a new algorithm (ALNOVE) proposed to dismiss redundant/noninformative items from the tool [31].

#### 2.4.4. The Multilingual Assessment Instrument for Narratives (MAIN)

The Greek version of the Multilingual Assessment Instrument for Narratives (MAIN) [32] was used to evaluate production of narrative skills at the macro- and microstructure levels. In this study, the “Baby Goats” story, a story similar in concept to an Aesop fable, making it suitable for adult populations, was used.

#### 2.4.5. The Raven's Coloured Progressive Matrices (RCPMs)

The 36-item Raven's Coloured Progressive Matrix (RCPM) [33] was applied for problem-solving ability examination.

### 2.5. Quality of Life Measure: Used at Baseline and at Follow-Up

#### 2.5.1. Stroke and Aphasia Quality of Life Scale-39 Item (SAQOL-39)

The Greek version of the Stroke and Aphasia Quality of Life scale-39 item (SAQOL-39) [34] was applied for the assessment of TMS effects on QoL. The Greek generic SAQOL-39 (SAQOL-39g) (i.e., the tool used in stroke patients without aphasia) is valid and reliable [35] and was used, and QoL was assessed using proxy ratings (caregivers) with all participants as three participants (P1, P4, and P5) struggled to respond to complex questions due to comprehension deficits.

### 2.6. Linguistic, Cognitive, and QoL Assessment and Analysis Procedures

**Figure 2:**
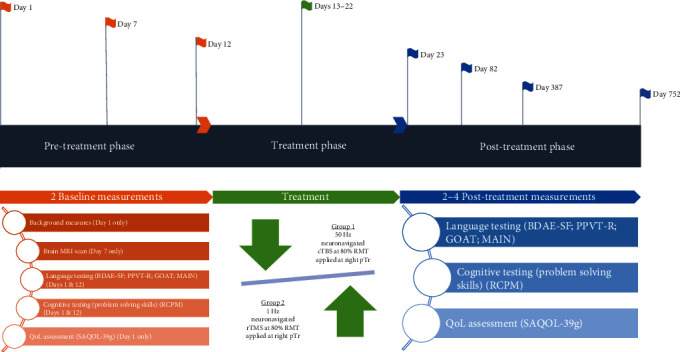
Experimental timeline of the study.

All participants were assessed twice at baseline, one day posttreatment, and at two-month follow-up on all cognitive-linguistic measures. One participant (P1) was further assessed one and two years posttreatment. A schematic diagram illustrating the experimental timeline is shown in Figure 2. All participants did a brain MRI a week before therapy initiation. To ensure treatment fidelity, the Template for Intervention Description and Replication (TIDieR) [36] was used. A speech-language pathologist, blind to the study, performed all assessments and recorded the results in the project database. A second speech-language pathologist, also blind to the study, analyzed the responses. For the analysis of the MAIN, the Quantitative Production Analysis (QPA) protocol [37] as adopted for Greek [38] was applied by a linguist blind to the study protocol.

### 2.7. Repetitive TMS (rTMS) Procedures and Protocol

The six participants were randomly (via a computer-generated randomization schedule) allocated to two groups (three participants in each group) with each group (T1 or T2) receiving only one treatment type. To minimize placebo effects, the participants were informed that they had 50% chance to receive real treatment and 50% chance to receive sham treatment. Therefore, they were blinded to their status of TMS conditioning (real vs. sham) until the end of the study. The treatment procedures that followed are described below.

### 2.8. Assessment of Resting Motor Threshold (RMT)

The assessment of rest motor thresholds (RMTs) needed for determination of stimulation intensity was carried out for each participant using surface electromyography (EMG) [39]. After locating the “hot spot,” for the appropriate RMT of the FDI, the standard stimulus magnitude used for mapping of the FDI was used and then the stimulus intensity was progressively reduced in 2% or 5% steps until the minimum single-pulse stimulator output intensity resulting in motor evoked potentials (MEPs) of at least 50 *μ*V peak-to-peak amplitude in ≥50% of pursued trials was found. The rate of stimulation was more than 3 secs between consecutive stimuli. Motor threshold levels were used to determine stimulation parameters as they were considered as an indication of cortical excitability.

### 2.9. Repetitive TMS (rTMS) Stimulation Parameters

Participants underwent rTMS at 80% of their individual RMT, using the Magstim Rapid2® stimulator (Magstim Co., Wales, UK) connected to a 70 mm Double Air Film Coil. Stimulation parameters were in accordance with published guidelines [40]. The position of the coil was guided by a frameless stereotactic neuronavigation system (ANT NEURO) that uses the individual patients' MRI scan to precisely localize the target area for stimulation. Before stimulation, a T1-weighted MRI image was obtained from each patient to locate the optimal coil position.

#### 2.9.1. Group T1: Continuous Theta Burst Stimulation (cTBS) over the Right Pars Triangularis (pTr)

Participants in this group (P1, P2, and P3) received inhibitory rTMS (continuous theta burst stimulation paradigm, cTBS) to the pTr in the right inferior frontal gyrus (homologous BA45), following a published protocol [18].

#### 2.9.2. Group T2: 1 Hz (Low Frequency) rTMS over the Right Pars Triangularis (pTr)

Participants in this group (P4, P5, and P6) received 10 daily stimulation treatments of 1 Hz rTMS (1200 pulses in 20 minutes each) over the right pTr.

### 2.10. Statistical Analyses

To analyze data with categorical outcomes (e.g., correct/incorrect and target word naming), Weighted Statistics (WEST) (“West-Trend” and “West-ROC” (one tailed)) were applied (see [41] for a review and the algorithm that calculated the weighted factors). Such statistics offer a mean of analyzing single-case study data when multiple baselines have been undertaken. Functional language data are reported in detail according to the QPA protocol, and QoL findings are reported rounded to two decimal places.

## 3. Results

### 3.1. Categorical Language and Cognitive Outcomes

**Figure 3:**
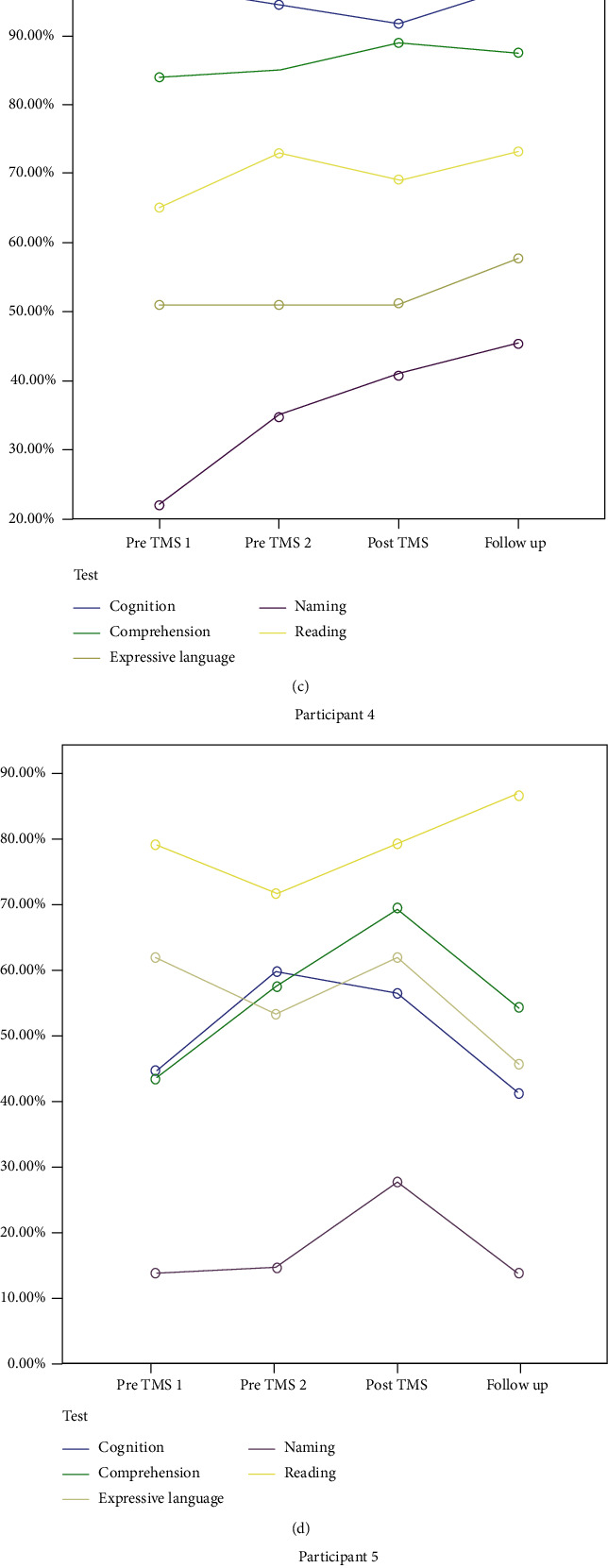
Short-term (one day posttreatment) and long-term (two months, one year, and two years posttreatment) effects of cTBS on cognitive and language performance for all 6 participants. The *Y* axis depicts relative values to demonstrate the magnitude of variation, if any, between assessments for each domain. Key: pre-TMS 1: baseline 1; pre-TMS 2: baseline 2; post-TMS: 1 day posttreatment; follow-up 1: 2 months posttreatment; follow-up 2: 1 year posttreatment; follow-up 3: 2 years posttreatment; follow-up: 2 months posttreatment.

The interrater reliability agreement between the two speech and language pathologists who analyzed the data was above 95%. The weights used in this study for the testing schedule of baseline 1, baseline 2, posttreatment (i.e., one day posttreatment), and follow-up (i.e., two months posttreatment) were as follows: (i) −3, −1, 1, and 3 in order to evaluate the trend across the study (WEST-Trend) and (ii) 3, −4, −1, and 2 to compare the rates of change (ROCs) across treatment and no treatment phases (WEST-ROC). This was the main analysis for all participants (P1-P6). However, for participant 1 (P1), the testing schedule was different: baseline 1, baseline 2, posttreatment (i.e., one day posttreatment) follow-up 1 (i.e., two months posttreatment), follow-up 2 (i.e., one year posttreatment), and follow-up 3 (i.e., two years posttreatment). The WEST-Trend and WEST-ROC weights up to follow-up 2 period were −2, −1, 0, 1, and 2 and 2, −2, −1, 0, and 1, respectively. The WEST-Trend and WEST-ROC weights for periods follow-up 1, follow-up 2, and follow-up 3 were −2, 0, and 2 and 1, −2, and 1, respectively. In the last two analyses (up to follow-up 2 and follow-up 3 stages) for P1, WEST-ROC evaluated the rates of change in the short versus long-term periods to explore the possible long-term (i.e., one and two years posttreatment) effects of TMS therapy. Performance on categorical language and cognitive data for all participants is reported in Tables 2 and 3 and Figure 3. Data relating to short-term and long-term effects (up to one-year follow-up) for participant 1 (P1) have been also published previously [42].

#### 3.1.1. Participant 1 (P1)

*(1) Short-Term Effects (i.e., One Day Posttreatment) of cTBS (Pre-TMS 1–Pre-TMS 2–Post-TMS)*. P1 did not show any overall improvement in comprehension (*t*(63) = 0.44, *p* = .32), problem-solving skills, naming, or reading. However, she showed moderate improvement in expressive language (*t*(25) = 1.79, *p* = .04), but this improvement was not higher in the treated (i.e., TMS period) versus the untreated periods (i.e., baseline periods) (*t*(25) = 0.90, *p* = .19).

*(2) Long-Term Effects (i.e., Two Months Posttreatment) of cTBS (Pre-TMS 2–Post-TMS–Follow-Up 1)*. P1 did not show any overall improvement in expressive language (*t*(25) = 0.57, *p* = .28), problem-solving skills, or naming. However, she showed significant improvement in comprehension (*t*(63) = 3.66, *p* < .001) and moderate improvement in reading (*t*(28) = 1.79, *p* = .04), and such improvements were greater during the first follow-up period (i.e., two months post-TMS) compared to the short-term (i.e., one day post-TMS) for both language comprehension (*t*(63) = 2.61, *p* < .01) and reading (*t*(28) = 1.79, *p* = .04).

*(3) Long-Term Effects (i.e., One Year Posttreatment) of cTBS (Post-TMS–Follow-Up 1–Follow-Up 2)*. P1 did not improve in expressive language (*t*(25) = 0.76, *p* = .75), cognition, and naming. However, she sustained significant improvement in comprehension (*t*(63) = 2.80, *p* = .003) and moderate improvement in reading (*t*(28) = 2.11, *p* = .02) up to one year post-TMS.

*(4) Long-Term Effects (i.e., Two Years Posttreatment) of cTBS (Follow-Up 1–Follow-Up 2–Follow-Up 3)*. P1 did not show any downward trend in cognition (*t*(35) = 1, *p* = .16), expressive language abilities (*t*(63) = 0, *p* = .5), naming, comprehension (*t*(63) = 0, *p* = .5), and reading (*t*(28) = −1, *p* = .84), showing that language gains in comprehension and reading were sustained at least up to two years posttreatment.

#### 3.1.2. Participant 2 (P2)

*(1) Short-Term Effects (i.e., One Day Posttreatment) of cTBS (Pre-TMS 1–Pre-TMS 2–Post-TMS)*. P2 did not show any overall improvement in either cognition (problem-solving skills) (*t*(35) = 0.32, *p* = .37), comprehension (*t*(63) = 1.52, *p* = .07), expressive language (*t*(25) = 0.46, *p* = .32), or naming (*t*(33) = −0.81, *p* = .79). However, he showed an overall improvement in reading (*t*(28) = 1.79, *p* = .04), but this improvement was not higher in the treated (i.e., TMS period) versus the untreated periods (i.e., baseline periods) (*t*(28) = 0.91, *p* = .187).

*(2) Long-Term Effects (i.e., Two Months Posttreatment) of cTBS (Pre-TMS 2–Post-TMS–Follow-Up 1)*. P2 did not show any overall improvement in either cognition (problem-solving skills) (*t*(35) = 0.37, *p* = .35), expressive language (*t*(25) = 0.63, *p* = .27), or reading (*t*(28) = 0.81, *p* = .21). However, he showed an overall improvement in comprehension (*t*(63) = 1.76, *p* = .041) and naming (*t*(33) = 1.75, *p* = .04), but such improvements were not higher during the first follow-up period (i.e., two months post-TMS) compared to short-term (i.e., one day post-TMS) for either comprehension (*t*(63) = 0.12, *p* = .45) or naming (*t*(33) = 1.07, *p* = .14).

#### 3.1.3. Participant 3 (P3)

*(1) Short-Term Effects (i.e., One Day Posttreatment) of cTBS (Pre-TMS 1–Pre-TMS 2–Post-TMS)*. P3 did not show any overall improvement in cognition (problem-solving skills) (*t*(35) = −1.43, *p* = .91), comprehension (*t*(63) = 1.13, *p* = .13), expressive language, or reading (*t*(28) = 1, *p* = .17). However, he showed an overall improvement in naming (*t*(33) = 3.01, *p* < .01), but this improvement was not higher in the treated (i.e., TMS period) versus the untreated periods (i.e., baseline periods) (*t*(33) = −.55, *p* = .71).

*(2) Long-Term Effects (i.e., Two Months Posttreatment) of cTBS (Pre-TMS 2–Post-TMS–Follow-Up 1)*. P3 did not show any overall improvement in cognition (problem-solving skills) (*t*(35) = 0.57, *p* = .28), comprehension (*t*(63) = 0.33, *p* = .37), expressive language (*t*(25) = 0.33, *p* = .37), naming (*t*(33) = 1.22, *p* < .01), or reading (*t*(28) = 0, *p* = .50).

#### 3.1.4. Participant 4 (P4)

*(1) Short-Term Effects (i.e., One Day Posttreatment) of 1 Hz rTMS (Pre-TMS 1–Pre-TMS 2–Post-TMS)*. P4 did not show any overall improvement in cognition (problem-solving skills) (*t*(35) = 1.07, *p* = .14), expressive language (*t*(25) = 0, *p* = .50), or reading (*t*(28) = 0, *p* = .50). However, she showed an overall improvement in comprehension (*t*(63) = 3.37, *p* < .001) and naming (*t*(33) = 2.31, *p* = 0.01), but this improvement was not higher in the treated (i.e., TMS period) versus the untreated periods (i.e., baseline periods) for either comprehension (*t*(63) = −.13, *p* = .55) or naming (*t*(25) = 1.09, *p* = .14).

*(2) Long-Term Effects (i.e., Two Months Posttreatment) of 1 Hz rTMS (Pre-TMS 2–Post-TMS–Follow-Up 1)*. P4 did not show any overall improvement in cognition (problem-solving skills) (*t*(35) = −2.23, *p* = .98), comprehension (*t*(63) = −.046, *p* = .67), expressive language (*t*(25) = −1, *p* = .83), naming (*t*(33) = −0.29, *p* = .61), or reading (*t*(28) = 1.44, *p* = .08).

#### 3.1.5. Participant 5 (P5)

*(1) Short-Term Effects (i.e., One Day Posttreatment) of 1 Hz rTMS (Pre-TMS 1–Pre-TMS 2–Post-TMS)*. P5 did not show any overall improvement in cognition (problem-solving skills) (*t*(35) = 0.43, *p* = .33), comprehension (*t*(63) = 0.46, *p* = .32), expressive language, naming, or reading (*t*(28) = 1.36, *p* = .09).

*(2) Long-Term Effects (i.e., Two Months Posttreatment) of 1 Hz rTMS (Pre-TMS 2–Post-TMS–Follow-Up 1)*. P5 did not show any overall improvement in cognition (problem-solving skills) (*t*(35) = 1, *p* = .16), expressive language, naming, or reading (*t*(28) = 0, *p* = .50). However, he showed an overall improvement in comprehension (*t*(63) = 2.72, *p* < .01), but such improvement was not higher during the first follow-up period (i.e., two months post-TMS) compared to short-term (i.e., one day post-TMS) (*t*(63) = 1.15, *p* = .12).

#### 3.1.6. Participant 6 (P6)

*(1) Short-Term Effects (i.e., One Day Posttreatment) of 1 Hz rTMS (Pre-TMS 1–Pre-TMS 2–Post-TMS)*. P6 did not show any overall improvement in cognition (problem-solving skills) (*t*(35) = 0, *p* = 0.5), expressive language (*t*(25) = 0.70, *p* = .25), or naming (*t*(33) = 0.37, *p* = .35). However, he showed an overall improvement in comprehension (*t*(63) = 2.60, *p* < .001) and reading (*t*(28) = 2.25, *p* = .02), but such improvements were not higher in the treated (i.e., TMS period) versus the untreated periods (i.e., baseline periods) for either comprehension (*t*(63) = 0.77, *p* = .21) or reading (*t*(28) = −0.15, *p* = .44).

*(2) Long-Term Effects (i.e., Two Months Posttreatment) of 1 Hz rTMS (Pre-TMS 2–Post-TMS–Follow-Up 1)*. P6 did not show any overall improvement in cognition (problem-solving skills) (*t*(35) = 1, *p* = .16), expressive language (*t*(25) = 1, *p* = .16), naming (*t*(33) = 1.49, *p* = .07), or reading (*t*(28) = .44, *p* = .33). However, he showed an overall improvement in comprehension (*t*(63) = 1.69, *p* = .04), but such improvement was not higher during the first follow-up period (i.e., two months post-TMS) compared to short-term (i.e., one day post-TMS) (*t*(63) = −1.58, *p* = .93).

### 3.2. Functional Language Outcomes

P1 and P5 had global aphasia and did not produce any narratives. A baseline average score was calculated for each linguistic index for each of the remaining participants (P2, P3, P4, and P6) individually. But for this study analyses, both baseline measurements were taken into account as they provided information on the range of microstructure performance.

#### 3.2.1. Participant 2 (P2)

*(1) Short-Term Outcomes (i.e., One Day Posttreatment)*. P2 produced a significantly higher number of narrative words (mostly adverbs and verbs) in the posttreatment assessment phase compared to baseline. Sentence productivity remained stable, and grammatical accuracy remained stable with an exception in the proportion of sentences with verbs that increased. The number and types of errors also remained stable. Results from the short-term microstructure analysis of the MAIN for P2 are shown in Table 4.

*(2) Long-Term Outcomes (i.e., Two Months Posttreatment)*. At follow-up, P2 reverted to baseline with regard to the total number of narrative words. This was the case for all lexical categories except pronouns that increased. Sentence productivity also remained stable. With regard to grammatical accuracy, the proportion of sentences with verbs reverted to baseline and well-formed utterances showed a downward trend. The number and types of errors remained stable. Results from the long-term microstructure analysis of the MAIN for P2 are shown in Table 4.

#### 3.2.2. Participant 3 (P3)

*(1) Short-Term Outcomes (i.e., One Day Posttreatment)*. P3 produced the same number of narrative words in the posttreatment assessment period compared to baseline. Sentence productivity remained stable. Grammatical accuracy also remained stable with the exception of the proportion of well-formed utterances that showed trends for improvement. With regard to error types and numbers, phonological errors and neologism showed a decreasing trend. Results from the short-term microstructure analysis of the MAIN for P3 are shown in Table 5.

*(2) Long-Term Outcomes (i.e., Two Months Posttreatment)*. At follow-up, P3 produced more narrative words (closed class words and nouns) compared to baseline. Sentence productivity remained stable, but the embedding index showed a declining trend. With regard to grammatical accuracy, the participant showed trends for improvement in the proportion of sentences with verbs and the proportion of well-formed utterances remained increased compared to baseline. With regard to error types and numbers; the number of phonological errors reverted to baseline, but neologisms retained the downward trend that was also exhibited in the short-term. Overall, the percentage of errors retained the downward trend that was also exhibited in the short-term. Results from the long-term microstructure analysis of the MAIN for P3 are shown in Table 5.

#### 3.2.3. Participant 4 (P4)

*(1) Short-Term Outcomes (i.e., One Day Posttreatment)*. No differences in the number of narrative words in the short-term were observed with the exception of closed class words that showed an upward trend. With regard to sentence productivity, MLU showed a trend of increase. In terms of grammatical accuracy, the proportion of well-formed utterances showed a declining trend, but no single word utterances were produced. As for error types and numbers, the participant made more phonological and lexical errors. Results from the short-term microstructure analysis of the MAIN for P4 are shown in Table 6.

*(2) Long-Term Outcomes (i.e., Two Months Posttreatment)*. At follow-up, P4 produced an overall lower number of narrative words compared to baseline. This was the case for closed class words and nouns. On the other hand, she produced more prepositions compared to baseline. Sentence productivity was similar to that of baseline. The overall percentage of errors and error types reverted to baseline. Results from the long-term microstructure analysis of the MAIN for P4 are shown in Table 6.

#### 3.2.4. Participant 6 (P6)

*(1) Short-Term Outcomes (i.e., One Day Posttreatment)*. P6 produced a higher number of narrative words in the posttreatment assessment compared to baseline. This was mainly the case for closed class words and adjectives. Sentence productivity increased significantly mainly in the MLU. Grammatical accuracy remained stable in all aspects except for the proportion of well-formed utterances that showed a declining trend. The proportion and types of errors remained stable. Results from the short-term microstructure analysis of the MAIN for P6 are shown in Table 7.

*(2) Long-Term Outcomes (i.e., Two Months Posttreatment)*. At follow-up, P6 retained the increasing trend he exhibited in the short-term with regard to the number of narrative words. This was mainly the case for closed class words, adjectives, and prepositions. Regarding sentence productivity, MLU and embedding indices remained increased compared to baseline, while the elaboration index decreased. Grammatical accuracy and the proportion and types of errors remained stable. Results from the long-term microstructure analysis of the MAIN for P6 are shown in Table 7.

### 3.3. Quality of Life Outcomes

Quality of life was assessed once at baseline (i.e., day 1 of study) and at follow-up (i.e., two months posttreatment) in all participants. However, P1 was further assessed at one- and two-year follow-ups. Results from the SAQOL-39g assessment are rounded to two decimal places and reported in Table 8. The observed score fluctuations in QoL domains for P1, P4, P5, and P6 were insignificant. However, P2 showed a moderate decline in the communication score and moderate-significant decline in the psychosocial score at two months posttreatment. P3 showed a moderate decline in the psychosocial score at two months posttreatment.

## 4. Discussion

This study set out to investigate the effectiveness of two rTMS paradigms (i.e., 1 Hz rTMS and cTBS) as standalone treatments for chronic poststroke aphasia in six individuals. Acute and subacute aphasia were both excluded from this study since only in chronic aphasia is there a remarkable slowing in the rate of spontaneous functional recovery [43].

The rationale behind the decision of using rTMS as a standalone treatment was based on (i) previous evidence suggesting that rTMS alone can lead to long-term language recovery in chronic aphasia poststroke [44–46] and (ii) the inconsistencies in SALT approaches (type and intensity) amongst several TMS aphasia studies [3–8, 20–23].

The decision to use two rTMS paradigms (i.e., cTBS and 1 Hz rTMS) was made in order to explore whether such protocols would induce similar changes in language performance in the sample under investigation, since both neuromodulation paradigms exert the same neurophysiological effects on the human brain (i.e., suppression of neuronal activity) even though they differ in the duration of TMS conditioning.

The trial followed a single study experimental design (SSED) in which all participants underwent two baseline measurements, then received 10 daily sessions of rTMS, and were reassessed one day and two months posttreatment. Participant 1 was further reassessed one- and two years posttreatment. The rest of the participants were not reassessed after the two-month follow-up period for several reasons (P3, P4, and P5 started one-to-one speech therapy; P2 lost interest as he did not observe any functional improvement; P6 started group aphasia therapy). More recently, it was alleged that two pretherapy probes can track the level of performance and rate of change [41]. In the present study, two baseline measurements were applied to lessen concerns that the observed effects may be due to random variation in subject performance and also to minimize placebo effects [42]. Furthermore, participants were blind to their status of TMS conditioning (real vs. sham) until the end of the study. Crucially, none of the six participants experienced any side effects during or after TMS conditioning.

Results from the present study corroborate findings from other studies that have successfully used TBS paradigms [13, 14, 16] revealing that cTBS and 1 Hz rTMS bring about comparable changes in language performance. In the short-term (i.e., one day posttreatment), all participants but one (P5 with global aphasia) showed trends towards improvement in several language skills. In the long-term (i.e., two months posttreatment), three participants showed trends towards improvement in various language skills. All three participants with anomic aphasia exhibited trends of improvement in comprehension (one in the short-term, one in the long-term, and one in the short- and long-term); two showed trends of improvement in reading (one in the short-term and one in the short- and long-term), and two showed trends towards improvement in naming (one in the short-term and one in the long-term). One participant (P1 with global aphasia) showed overall improvements in comprehension and reading at two months and at one-year follow-up [42] that were sustained two years posttreatment as well. Notably, this was the oldest participant who exhibited severe global aphasia resulting from diffuse left hemispheric lesions that also had the least years of education (i.e., six) compared to the other participants. No decline in linguistic and cognitive performance compared to baseline was observed in any participant. Also, none of the participants showed any (trend towards) improvement in the control variable (i.e., problem-solving skills). The control variable was assessed at baseline as many times (i.e., two) as the dependent language variables (i.e., comprehension, expression, reading, and naming accuracy) in all participants, and as it remained stable in all participants, it was assumed that (i) the chances that TMS led to language specific gains were increased and (ii) the possibilities for the placebo and training effects were reduced.

To date, three studies have shown that 1 Hz (LF) rTMS as a standalone therapy can lead to language gains in some PWA. In particular, one study [44] investigated the effects of 1 Hz rTMS on naming performance and noticed immediate and long-lasting improvements (6 months posttreatment) in nine individuals with mild-to-moderate chronic nonfluent aphasia. In the present study, along with two participants that had anomic aphasia and exhibited trends towards improvement in naming, the participant with Broca's aphasia also showed a trend of improvement in naming, however only in the short-term. In another study, improvements in several language skills (i.e., naming, repetition, picture description tasks, and length of utterances) were observed that lasted up to 12 months post (1 Hz)-rTMS in six people with chronic nonfluent aphasia poststroke [45]. In the present study, one participant with severe global aphasia showed sustained improvements in comprehension and reading two months, one year, and two years posttreatment. In another trial [46], an increase in the number of closed-class words of discourse productivity was noticed in 10 individuals with chronic nonfluent aphasia two months posttreatment with 1 Hz rTMS. In our study, the analysis of narratives yielded mixed results. With regard to error types and percentages, the participant with Broca's aphasia (P3) exhibited less phonological errors and neologisms in the short-term and less neologisms in the long-term. On the other hand, one of the participants with moderate-severe anomic aphasia (P4) made more phonological and lexical errors in the short-term but reverted to baseline performance in the long-term. Discourse productivity increased in the short-term in one participant with moderate-severe anomic aphasia (P2) and in the long-term in the participant with Broca's aphasia (P3). The participant with mild anomic aphasia (P6) showed improvement in the short-term that was also sustained in the long-term. Finally, one of the participants with moderate-severe anomic aphasia (P4) showed a declining trend only in the long-term. Interestingly, the participant with mild anomic aphasia (P6) manifested an increase in his MLU both in the short- and long-term.

Up until now, several TMS randomized controlled trials (RCTs) have indicated that 1 Hz rTMS over the contralesional IFG in conjunction with SALT has the potential to drive change in various language domains in at least some people with subacute [3–5, 8, 22, 23] and chronic aphasia [11, 21]. Nonetheless, there are several inconsistencies in those studies concerning the site of stimulation within the homologue of Broca's area; the methods of localization of the stimulation site; the ingredients, dosage, and intensity of the adjunct SALT; the number and types of language outcomes measures; and the number and duration of follow-up assessments. In addition, not all studies have reported positive outcomes. For example, a most recent RCT [47] did not find any beneficial add on effects of 1 Hz rTMS to SALT in chronic poststroke aphasia rehabilitation. Another study raises concerns about applying LF TMS over the right pTr in patients with apraxia of speech (AoS) [48]. In this study, the researchers demonstrated that a 69-year-old individual with AoS due to a left first ever small ischemic stroke of the left precentral gyrus deteriorated after one session of real cTBS over the contralesional precentral gyrus and improved after sham cTBS over the same area according to both objective and subjective evaluations. The findings of those trials highlight the possible impact of lesion location on noninvasive neuromodulation response and point towards the development of individualized rTMS aphasia rehabilitation protocols by considering individual-intrinsic variables (age at the time of stroke, lesion volume and location, white matter integrity, and cognitive-linguistic impairment) and individual extrinsic variables (e.g., environment, treatment mode, language, and brain recovery) [49], rather than providing a “one-size fits all” neuromodulation approach. Furthermore, such findings imply that expressive language processes rely on cortical networks that involve both hemispheres.

In addition to RCTs supporting the potential benefit of LF rTMS on aphasia rehabilitation poststroke, some systematic reviews with/without meta-analyses are also supportive [50–53]. However, other recent work has indicated that the quality of the conduct of reviews 50-53 is low, and therefore, more research is needed [54]. More recently, a meta-analysis of RCTs and randomized cross-over trials [55] found a moderate long-term effect size of rTMS effects in language gains especially in naming in both subacute and chronic patients with aphasia. In this review, five studies applied LF rTMS, one study combined LF with HF rTMS, and one study compared LF with HF and sham TMS (see [55] and references within).

Overall, research in the field of 1 Hz rTMS to the contralesional IFG in aphasia recovery is ongoing but is also parallel to trials investigating the effects of different paradigms, either in terms of stimulation sites and/or TMS paradigms per se. For example, an emerging number of studies have started exploring inhibitory cTBS over the contralesional IFG [15, 25, 56], excitatory iTBS over perilesional areas of the left hemisphere [12, 13, 16], and sequential cTBS and iTBS [14].

Aphasia-related TMS research is flourishing, and TMS technology has now become a mainstream application in many aphasia labs worldwide. The challenge researchers are facing is the unravelment of the mechanisms of TMS-induced language recovery and the understanding of why some people respond (more or less) whilst others do not respond to this neuromodulation technique. Despite numerous clinical studies that have explored the therapeutic potential of rTMS in several neurological disorders, the cellular and molecular mechanisms responsible for the after-effects of rTMS are largely unknown. The mixture of LTD and LTP effects on synapses measured by MEP behavioral changes is highly variable across individuals, showing that it would be an oversimplification to describe the rTMS after-effects as LTD or LTP-like plasticity solely based on MEP modifications [57]. Additional research is needed to elucidate how structural and functional properties of individual neurons and local networks are related to the effects of single pulse rTMS [58]. Beyond the molecular mechanisms underlying behavioral recovery, a few insightful accounts about the underpinnings of the observed TMS-induced language improvement, that also explain the rationale behind the application of various aphasia TMS protocols, have been suggested and are based in principle on models of brain reorganization after a stroke.

The first account is related to stroke-induced disruption of the interhemispheric balance. This disruption leads to reduced inhibition from the affected to the unaffected hemisphere and to increased and deleterious inhibition of the affected from the unaffected hemisphere [59]. This process is considered maladaptive for language recovery as it blocks the dominant hemisphere, where language processes are established, from resuming their role in language processes [60]. The decision of applying LF rTMS over the contralesional hemisphere in this research was motivated by the hypothesis that by inhibiting the right hemisphere, residual language supported by the left hemisphere is released from transcallosal inhibitory input by the intact right hemisphere [61].

A second possible scenario is that language gains are associated with recruitment of regions of the right hemisphere that are homotopic to the damaged components of the left language network [62]. A third account is based on the increasingly accepted theory that language processes rely on highly localized brain regions and bilaterally distributed brain networks [63], and language reorganization poststroke is based on domain-specific and domain-general network processes [64]. The hypothesis that the suppression of a hyperactive right pTr with LF rTMS modulates the right pars opercularis (pOp), and in turn, other right brain regions may explain the results of the present study.

In addition to the unravelment of the neural mechanisms of TMS-induced language recovery, cognitive and psycholinguistic analyses demonstrating which cognitive processes are implicated in language facilitation and where in the language system, rTMS induces language improvements, may provide researchers with an insight into the issue of candidacy for and responsiveness to TMS. On this basis, research is poor as most clinical aphasia studies focus on the mechanistic aspects of recovery (i.e., neuroanatomical and behavioral changes). Some explanations however provide evidence on how the language system is reorganized post-TMS. It is postulated that the observed improvement in discourse productivity in chronic nonfluent aphasia may be explained by TMS-induced improved lexical-semantic access allowing retrieval of word- and word meaning representations [46]. This could explain the noticeable improvement in accessing words in several categories and no improvement in grammatical complexity or sentence construction [46]. In the present study, there was only one participant with nonfluent aphasia who showed improvement in discourse productivity in the long-term and the above account may explain his performance.

The current study has several strengths as follows. First, we suggest adopting an SSED methodology in aphasia research and using WEST statistics to measure treatment change as such statistics are suitable for studies with small numbers of participants and nonhomogenous profiles. Second, we performed follow-up assessments to investigate the long-term effects of TMS treatment. In fact, one participant was followed up for two years posttreatment and demonstrated sustained language gains in comprehension and reading skills in that period. The findings corroborate prior evidence that TMS can lead to sustained language changes without any additional behavioral therapy [44–46]. Third, this study employed an ecologically valid measure to assess functional communication which is related to phrase and sentence production and narration and not experimental language tasks. Finally, as stroke affects health-related QoL [65], the effects of treatment on the QoL of the participants were also assessed. Proxy ratings were used as three participants struggled to respond to questions due language comprehension problems. Existing evidence supports that proxies exaggerate QoL problems of patients [66]. Hence, caution is needed when proxies contribute to QoL assessments. Nonetheless, when patient reports cannot be obtained, proxies can be helpful [67]. In the current study, the findings indicated that QoL did not significantly change in three participants because of the treatment. For the remaining participants, posttreatment communication scores showed a declining trend in three participants and the psychological scores dropped for two others. Such findings clearly capture the difference between statistical and clinical significance. Statistical significance is important for researchers and service providers but is of little value to patients and their families. Clinical significance is vital for the person with communication problems and their caregivers. Based on the results from the QoL measure, treatment results failed to meet the needs and expectations of the participants and their families. Therefore, we strongly recommend that future TMS aphasia studies are also aimed at capturing the clinical significance of this type of treatment using relevant tools.

Despite the promising results of this study, there are several limitations that warrant discussion. First, the sample size was small, and the participants had various clinical profiles. This compromises the generalizability of our findings, but on the other hand, this clinical profile heterogeneity can be seen as advantageous as it is typical of what is observed in clinical settings. Also, the fact that the TMS protocol was the same for all participants (i.e., inhibitory rTMS) allowed insight into who may benefit more from this particular protocol. It seems, for example, that it could prove beneficial for global aphasia on the grounds of diffuse left hemisphere damage. However, as direct measurements of brain activation and connectivity were not obtained, no hypotheses could be formulated regarding which model(s) of brain-reorganization best explain(s) the findings. In TMS aphasia research, direct measures of brain activation and connectivity are needed to help with the elucidation of the neuroplastic effects of treatments [42]. Realistically though, individual fMRI localization is expensive, time consuming, and not available in all aphasia labs.

We suggest that to enhance the effectiveness of rTMS in aphasia rehabilitation, future studies should systematically document all their data in an Aphasia TMS Database similar to the PLORAS (predicting language outcome and recovery after stroke) project [68]. In particular, with regard to participants' intrinsic factors, parameters such as age, lesion location and size, vascular perfusion, brain connectivity and integrity of white matter, genetics, body mass indices, sex, handedness, education, type of aphasia, and its severity should all be documented as there is robust evidence that they all affect aphasia recovery. For instance, with regard to age, there is evidence that young people with aphasia improve more compared to older individuals [69]. Regarding lesion location, some studies suggest that lesions involving the left STG (superior temporal gyrus) and Wernicke's area are associated with poor aphasia improvement [70]. With regard to lesion magnitude, even though large left hemisphere lesions are typically associated with poorer recovery [71], in a recent study, patients with larger stroke volumes showed greater aphasia improvements regardless of the involvement of the language areas [72]. This could explain the findings from the current study in which the only participant who showed statistically significant improvements had diffuse and large brain lesions in the left hemisphere. In addition, several studies have shown shifts in vascular perfusions poststroke [73, 74], but the extent to which such alterations influence recovery of the neural networks for language is unknown [49]. Furthermore, the degree of white matter integrity in the infarcted hemisphere together with the integrity of white matter tracts in the contralesional hemisphere is also likely to be linked to recovery ([49] and references within). Research on the role of BDNF (brain-derived neurotrophic factor) variants on language recovery poststroke is emerging, and several studies have demonstrated that it influences recovery [75, 76]. Also, through univariate analysis, it has been shown that total lean body mass—not adipose tissue—may be a positive factor for predicting aphasia improvement [72]. Lastly, evidence in relation to the impact of sex [77], handedness, and educational background [78] on language recovery is controversial.

Aphasia severity has been shown to be a good predictor of recovery of both short- [79] and long-term outcomes [80]. It is postulated that all the above biological (intrinsic) factors have a synergistic effect on language recovery poststroke, and this can be verified by the observed variability in progression of aphasia and recovery even between people with the same type of aphasia. In addition, TMS parameters (e.g., type of coil, stimulation site, duration, dosage, intensity, and frequency of the stimulation) also affect outcomes. In particular, the amount of surface charge produced and thus the extent of action of the current in the brain tissue depend on many biological and physical parameters such as the magnetic pulse waveform, the intensity, frequency and pattern of stimulation, the type and orientation of coil, the distance between coil and brain, and the respective orientation of the current lines and excitable neuronal elements into the brain [43]. For example, if the handle of f8c is oriented parallel to the interhemispheric midline (posteroanterior direction), motor cortex TMS activates the pyramidal tract only indirectly through interneurons [81]. When the handle of an f8c is oriented perpendicular to the interhemispheric midline (lateromedial direction), both interneurons and pyramidal neurons are activated [82]. The lowest intensity threshold to elicit MEPs in the M1 is achieved when the stimulus creates a posteroanterior current that is orthogonal to the central sulcus (i.e., the handle of the f8c oriented 45° posteriorly and laterally) [83], but the reverse orientation (anteroposterior) makes the latency time increase by several milliseconds [43] and is considered better for inducing motor cortex plasticity [84]. To optimize the effects of TMS, it is suggested that the strength of the electric field perpendicular to the targeted area (for all cortical surface areas) is maximised [85]. Also, even though MEP measurements in healthy individuals have led to the consensus that low-frequency stimulation (≤1 Hz) induces inhibition, whereas high frequencies (≥ 5 Hz) induce excitation [43], both conditions may lead to mixed effects [86]. By doubling, for example, the duration of stimulation on the motor cortex inhibition may reverse to excitation and vice versa [87]. Moreover, SALT is the gold standard in aphasia rehabilitation [88], and the above discussion demonstrates the high variability and lack of standardization of SALT approaches in the field of aphasia rehabilitation [89]. The effectiveness of SALT approaches first needs to be evaluated against standards of evidence-based practice (EBP). Until then, researchers are prompted to use structured aphasia programs as adjuncts to TMS based on the evidence that this leads to neuroplastic changes that support aphasia recovery.

## 5. Conclusion

The advent of modern noninvasive brain stimulation techniques has shifted the attention of aphasia rehabilitation scientists to additional ways that could enhance plasticity in the lesioned language brain network. Even though the number of studies that have applied TMS in poststroke aphasia rehabilitation is increasing, results remain controversial. From the current findings, it can be concluded that inhibitory TMS over the right pTr has the potential to drive neuroplastic changes that facilitate language recovery in chronic poststroke aphasia. However, to elucidate the precise mechanisms of action that TMS exerts in the lesioned language network, researchers are urged to experiment with different protocols and follow up their participants for potential long-term and generalization effects. The importance of the clinical relevance of therapies urges future researchers to include ecological outcome measures that capture the effects of TMS aphasia treatment on everyday communication.

## Figures and Tables

**Table 1:** Demographics and clinical characteristics of the participants.

Participant	Sex	Age (years)	Handedness	Education (years)	Type of stroke	Months post stroke	Lesion site (left hemisphere)	Type of aphasia	Severity of aphasia	SALT prior to enrolment	Termination of SALT
1	F	74	Right	6	Ischemic	48	Diffuse frontal, parietal, and temporal (middle and superior gyri) lobes; insula; basal ganglia	Global	Severe	20 months–2 times per week–45 min of SALT	2 years before enrolment
2	M	61	Right	12	Ischemic	9	Broca's and Wernicke's areas; arcuate fasciculus; insula; inferior precentral gyrus; temporal pole	Anomic	Moderate-severe	6 months–2 times per week–45 min of SALT	2 months before enrolment
3	M	48	Right	15	Ischemic	11	IFG; internal capsule; insula; caudate nucleus; putamen; inferior precentral gyrus	Broca's	Moderate-severe	8 months–4 times per week–45 minutes	10 days before enrolment
4	F	72	Right	12	Ischemic	50	Broca's and Wernicke's areas; arcuate fasciculus; insula; superior posterior temporal gyrus; middle posterior temporal gyrus	Anomic	Moderate-severe	24 months–2 times per week–45 min of SALT	2 years before enrolment
5	M	55	Right	17	Ischemic	8	Precentral gyrus; postcentral gyrus; arcuate fasciculus; internal capsule; caudate nucleus; putamen	Global	Severe	4 months–4 times per week–45 minutes	10 days before enrolment
6	M	26	Right	16	Ischemic	109	IFG; MFG; SFG; insula; basal ganglia; arcuate fasciculus; internal capsule; anterior temporal lobe; Wernicke's area, anterior temporal lobe most likely due to an arachnoid cyst	Anomic	Mild	10 months–4 times per week–45 minutes	7 years before enrolment

**Table 2:** Categorical language and cognitive scores at posttreatment and follow-up compared to baseline for P1, P2, and P3.

Participant item	P1						P2				P3			
	B1	B2	P-TMS	F1	F2	F3	B1	B2	P-TMS	F1	B1	B2	P-TMS	F1
Problem-solving skills	7/36	8/36	8/36	8/36	7/36	7/36	27/36	30/36	28/36	32/36	35/36	34/36	33/36	35/36
Auditory comprehension	12/64	13/64	13/64	26/64	24/64	24/64	18/64	18/64	21/64	24/64	31/64	29/64	30/64	31/64
Expressive language (Boston naming test—excluded)	0.5/26	0.5/26	2/26	1/26	1/26	1/26	4/26	4/26	5/26	6/26	13.5/26	13.5/26	13.5/26	15/26
Naming—accuracy	1/34	0/34	1/34	0/34	1/34	1/34	4/34	2/34	2/34	6/34	17/34	12/34	14/34	15.5/34
Reading skills	2/29	2/29	2/29	5/29	6/29	4/29	14/29	14/29	17/29	16/29	14/29	19/29	18/29	19/29

Key: P1: participant 1; P2: participant 2; P3: participant 3; B1: baseline 1; B2: baseline 2; P-TMS: post-TMS (1 day posttreatment); F1: follow-up 1 (2 months posttreatment); F2: follow-up 2 (1 year posttreatment); F3: follow-up 3 (2 years posttreatment).

**Table 3:** Categorical language and cognitive scores at posttreatment and follow-up compared to baseline for P4, P5, and P6.

Participant item	P4				P5				P6			
	B1	B2	P-TMS	F1	B1	B2	P-TMS	F1	B1	B2	P-TMS	F1
Problem-solving skills	17/36	22/36	21/36	16/36	33/36	32/36	34/36	34/36	32/36	32/36	32/36	34/36
Auditory comprehension	30/64	38/64	45/64	36/64	15/64	14/64	17/64	27/64	46/64	48/64	55/64	53/64
Expressive language (Boston naming test—excluded)	16.5/26	14.5/26	16.5/26	12.5/26	0/26	0/26	0/26	0/26	23.5/26	24.5/26	24.5/26	28.5/26
Naming—accuracy	4.5/34	5/34	9/34	4.5/34	0/34	0/34	0/34	0/34	25/34	25.5/34	25.5/34	28.5/34
Reading skills	23/29	21/29	23/29	25/29	8/29	9/29	11/29	9/29	24/29	26/29	28/29	27/29

Key: P4: participant 4; P5: participant 5; P6: participant 6; B1: baseline 1; B2: baseline 2; P-TMS: post-TMS (1 day posttreatment); F1: follow-up 1 (2 months posttreatment).

**Table 4:** Short-term (one day posttreatment) and long-term (two months posttreatment) effects of cTBS on narration outcomes (i.e., functional communication) for participant 2.

Category	Participant 2				
Lexical selection	Pre-TMS 1	Pre-TMS 2	Baseline	Post-TMS	Follow-up
Closed class	10	21	15.50	20	10
Nouns	3	3	3.00	4	1
Adjectives	4	7	5.50	11	4
Prepositions	4	7	5.50	6	1
Adverbs	1	4	2.50	16	5
Pronouns	11	8	9.50	14	18
Verbs	18	21	19.50	31	18
Sentence productivity					
MLU	2.55	4.44	3.49	3.40	3.17
Elaboration index	1.06	1.75	1.40	1.21	1.53
Embedding index	0.00	0.31	0.16	0.10	0.17
Discourse productivity					
Narrative words	51	71	61.00	102	57
Grammatical accuracy					
Prop of S with V	18	16	17.00	29	15
Prop of U w/o V	2	0	1.00	0	2
Prop of single word U	0	0	0.00	1	1
Prop of well-formed U	0.94	0.75	0.85	0.79	0.33
AUX complexity index	1.06	1.07	1.06	1.00	1.00
Error types					
Phonological	1	1	1.00	2	2
Morphosyntactic	2	0	1.00	2	1
Semantic	1	0	0.50	0	1
Lexical	2	2	2.00	3	3
Neologisms	0	0	0.00	0	0
Circumlocution	0	1	0.50	0	1
Phonological %	0.02	0.01	0.02	0.02	0.04
Morphosyntactic %	0.04	0.00	0.02	0.02	0.02
Semantic %	0.02	0.00	0.01	0.00	0.02
Lexical %	0.04	0.03	0.03	0.03	0.05
Neologisms %:	0.00	0.00	0.00	0.00	0.00
Circumlocution %	0.00	0.01	0.01	0.00	0.02
All errors %	0.12	0.06	0.08	0.07	0.14

Key: prop: proportion; s: sentences; V: verbs; U: utterances; w/o: without.

**Table 5:** Short-term (one day posttreatment) and long-term (two months posttreatment) effects of cTBS on narration outcomes (i.e., functional communication) for participant 3.

Category	Participant 3				
Lexical selection	Pre-TMS 1	Pre-TMS 2	Baseline	Post-TMS	Follow-up
Closed class	21	22	21.50	25	33
Nouns	17	19	18.00	21	29
Adjectives	4	2	3.00	1	5
Prepositions	5	5	5.00	7	7
Adverbs	3	2	2.50	0	2
Pronouns	6	8	7.00	4	8
Verbs	19	19	19.00	19	23
Sentence productivity					
MLU	5.36	6.42	5.89	6.42	5.35
Elaboration index	2.31	2.92	2.61	2.67	2.25
Embedding index	0.36	0.58	0.47	0.58	0.15
Discourse productivity					
Narrative words	75	77	76.00	77	107
Grammatical accuracy					
Prop of S with V	13	12	12.50	12	20
Prop of U w/o V	1	0	0.50	0	0
Prop of single word U	0	0	0.00	0	0
Prop of well-formed U	0.38	0.42	0.40	0.75	0.60
AUX complexity index	1.07	1.00	1.04	1.17	1.05
Error types					
Phonological	26	25	25.50	21	28
Morphosyntactic	3	2	2.50	4	5
Semantic	0	1	0.50	0	0
Lexical	4	0	2.00	0	3
Neologisms	4	4	4.00	0	0
Circumlocution	0	0	0.00	0	2
Phonological %	0.35	0.32	0.34	0.27	0.26
Morphosyntactic %	0.04	0.03	0.03	0.05	0.05
Semantic %	0.00	0.01	0.01	0.00	0.00
Lexical %	0.05	0.00	0.03	0.00	0.03
Neologisms %	0.05	0.05	0.05	0.00	0.00
Circumlocution %	0.00	0.00	0.00	0.00	0.02
All errors %	0.49	0.42	0.45	0.32	0.36

Key: prop: proportion; s: sentences; V: verbs; U: utterances; w/o: without.

**Table 6:** Short-term (one day posttreatment) and long-term (two months posttreatment) effects of 1 Hz rTMS on narration outcomes (i.e., functional communication) for participant 4.

Category	Participant 4				
Lexical selection	Pre-TMS 1	Pre-TMS 2	Baseline	Post-TMS	Follow-up
Closed class	15	21	18.00	26	7
Nouns:	11	21	16.00	20	6
Adjectives	0	7	3.50	2	0
Prepositions	0	1	0.50	1	6
Adverbs	1	1	1.00	0	0
Pronouns	14	8	11.00	6	13
Verbs	11	17	14.00	12	10
Sentence productivity					
MLU	3.50	4.00	3.75	4.86	3.23
Elaboration index	2.38	1.53	1.95	1.64	1.60
Embedding index	0.14	0.05	0.10	0.07	0
Discourse productivity					
Narrative words	52	76	64,00	68	42
Grammatical accuracy					
Prop of S with V	8	15	11.50	11	10
Prop of U w/o V	3	2	2.50	3	1
Prop of single word U	3	2	2.50	0	2
Prop of well-formed U	0.50	0.27	0.38	0.09	0.50
AUX complexity index	1.00	1.00	1.00	0.90	1.00
Error types					
Phonological	0	2	1.00	6	1
Morphosyntactic	3	14	8.50	14	2
Semantic	0	5	2.50	4	3
Lexical	0	1	0.50	3	2
Neologisms	1	1	1.00	0	1
Circumlocution	0	0	0.00	1	0
Phonological %	0.00	0.03	0.02	0.09	0.02
Morphosyntactic %	0.06	0.18	0.13	0.21	0.05
Semantic %	0.00	0.07	0.04	0.06	0.07
Lexical %	0.00	0.01	0.01	0.04	0.05
Neologisms %	0.02	0.01	0.02	0.00	0.02
Circumlocution %	0.00	0.00	0.00	0.01	0.00
All errors %	0.08	0.30	0.21	0.41	0.21

Key: prop: proportion; s: sentences; V: verbs; U: utterances; w/o: without.

**Table 7:** Short-term (one day posttreatment) and long-term (two months posttreatment) effects of 1 Hz rTMS on narration outcomes (i.e., functional communication) for participant 6.

Category	Participant 6				
Lexical selection	Pre-TMS 1	Pre-TMS 2	Baseline	Post-TMS	Follow-up
Closed class	22	30	26.00	41	41
Nouns	17	26	21.50	27	24
Adjectives	3	3	3.00	10	12
Prepositions	6	6	6.00	8	13
Adverbs	3	3	3.00	3	2
Pronouns	4	5	4.50	4	3
Verbs	14	21	17.50	23	22
Sentence productivity					
MLU	6.56	6.00	6.28	9.67	8.83
Elaboration index	3.33	2.93	3.13	4.17	1.83
Embedding index	0.5	0.38	0.44	0.92	0.85
Discourse productivity					
Narrative words	69	94	81.5	116	117
Grammatical accuracy					
Prop of S with V	9	15	12	11	12
Prop of U w/o V	1	1	1	1	1
Prop of single word U	0	0	0	0	0
Prop of well-formed U	0.89	0.93	0.91	0.73	0.83
AUX complexity index	1.11	1.07	1.09	1.00	1.00
Error types					
Phonological	4	0	2.00	0	0
Morphosyntactic	3	0	1.50	1	2
Semantic	0	3	1.50	0	4
Lexical	1	2	1.50	2	2
Neologisms	0	0	0.00	0	0
Circumlocution	0	0	0.00	1	0
Phonological %	0.06	0.00	0.02	0.00	0.00
Morphosyntactic %	0.04	0.00	0.02	0.01	0.02
Semantic %	0.00	0.03	0.02	0.00	0.03
Lexical %	0.01	0.02	0.02	0.02	0.02
Neologisms %	0.00	0.00	0.00	0.00	0.00
Circumlocution %	0.00	0.00	0.00	0.01	0.00
All errors %	0.12	0.05	0.08	0.03	0.07

Key: prop: proportion; s: sentences; V: verbs; U: utterances; w/o: without.

**Table 8:** Quality of life at the pretreatment (baseline) stage and at 2 months follow-up using the SAQOL-39g for all participants and at baseline, at 2 months follow-up, at 1 year follow-up, and at 2 years follow-up for participant 1.

Item (maximum score: 5)	Participant 1				Participant 2		Participant 3		Participant 4		Participant 5		Participant 6	
	Baseline	2 months post-TMS	1 year post-TMS	2 years post-TMS	Baseline	2 months post-TMS	Baseline	2 months post TMS	Baseline	2 months post TMS	Baseline	2 months post TMS	Baseline	2 months post TMS
SAQOL-39g mean score	2.05	2.18	2.12	1.9	3.57	3.1	4.03	3.82	3.32	3.2	1	1	4.24	4.28
Physical score	2.38	2.44	2.25	2.1	4.62	4.43	5	5	4.68	4.5	1	1	4.75	4.81
Communication score	1.57	1.72	1.85	1.6	2.71	2.28	3.28	3.14	2.42	2.1	1	1	4.71	4.71
Psychosocial score	2.2	2.38	2.25	2	3.37	2.56	3.81	3.31	2.87	3	1	1	3.25	3.31

## Data Availability

The data of the present study are available from the corresponding author upon rational request.
